# Lipids associated with autophagy: mechanisms and therapeutic targets

**DOI:** 10.1038/s41420-024-02224-8

**Published:** 2024-10-30

**Authors:** Michał Jarocki, Kacper Turek, Jolanta Saczko, Mounir Tarek, Julita Kulbacka

**Affiliations:** 1 University Clinical Hospital, Wroclaw Medical University, Wroclaw, Poland; 2 Provincial Specialist Hospital, Wroclaw, Poland; 3 Department of Molecular and Cellular Biology, Faculty of Pharmacy, Wroclaw Medical University, Wroclaw, Poland; 4 Université de Lorraine, CNRS, LPCT, Nancy, France; 5 Department of Immunology and Bioelectrochemistry, State Research Institute Centre for Innovative Medicine, Vilnius, Lithuania

**Keywords:** Mitophagy, Macroautophagy

## Abstract

Autophagy is a molecular process essential for maintaining cellular homeostasis, with its impairment or dysregulation linked to the progression of various diseases in mammals. Specific lipids, including phosphoinositides, sphingolipids, and oxysterols, play pivotal roles in inducing and regulating autophagy, highlighting their significance in this intricate process. This review focuses on the critical involvement of these lipids in autophagy and lipophagy, providing a comprehensive overview of the current understanding of their functions. Moreover, we delve into how abnormalities in autophagy, influenced by these lipids, contribute to the pathogenesis of various diseases. These include age-related conditions such as cardiovascular diseases, neurodegenerative disorders, type 2 diabetes, and certain cancers, as well as inflammatory and liver diseases, skeletal muscle pathologies and age-related macular degeneration (AMD). This review aims to highlight function of lipids and their potential as therapeutic targets in treating diverse human pathologies by elucidating the specific roles of phosphoinositides, sphingolipids, and oxysterols in autophagy.

## Facts

- Phosphoinositides, Sphingolipids, and Oxysterols: These lipids have significant roles in regulating autophagy, thus emphasizing their critical involvement in the process of cellular homeostasis.
- Lipids and Disease Progression: Dysregulation of autophagy, influenced by these specific lipids, contributes to a broad range of diseases, including cardiovascular disorders, neurodegenerative conditions, type 2 diabetes, certain cancers, and inflammatory diseases.
- Therapeutic Potential: Targeting the specific roles of phosphoinositides, sphingolipids, and oxysterols in autophagy may offer promising therapeutic strategies for treating various diseases.

## Open Questions

- What are the exact molecular mechanisms by which phosphoinositides, sphingolipids, and oxysterols regulate autophagy and lipophagy?
- How does the dysregulation of lipid-mediated autophagy specifically affect the progression of each major disease type, and what variations exist across different conditions?
- What are the challenges and potential methods for developing targeted therapies that modulate lipid-regulated autophagy pathways without causing significant off-target effects?
- In what ways does aging influence the lipid-autophagy relationship, particularly in age-related diseases like AMD and skeletal muscle pathologies?

## Introduction

Autophagy is an essential catabolic process in cellular function that plays a significant role in the maintenance of homeostasis. Autophagy was initially considered a housekeeping mechanism, allowing a cell to dispose of damaged organelles and proteins, but recent studies indicate that it also promotes the survival of a cell exposed to unfavorable conditions [1]. These include starvation of amino acids and energy, exposure to reactive oxygen species (ROS), lack of growth factors, and general stress [2]. Among other functions, autophagy allows a cell to degrade damaged proteins, foreign pathogens, aged organelles, and other cytosolic components [3]. Thus, autophagy can be selective and target specific, preselected components of cytoplasm or nonselective when large parts of cytoplasm are degraded, usually in response to general stress. Lipophagy, a form of autophagy that targets lipid droplets (LDs) is an example of selective autophagy [4]. Although there are 3 distinctive types of autophagy (macro-autophagy, micro-autophagy, and chaperone-mediated autophagy), they all share a common end stage of lysosomal degradation of cellular structures and proteins [5]. Microautophagy is a process in which small molecules are engulfed by the lysosomal membrane itself [6]. Chaperone-mediated autophagy (CMA) proteins containing the KFERQ motif - a recognition template- are recognized and translocated across the lysosomal membrane by LAMP-2A protein [7]. The process of macroautophagy (herein referred to as autophagy) can be divided into 5 stages: initiation, elongation, maturation, fusion and degradation [8]. Various stimuli trigger a complex autophagy machinery during the initiation, resulting in phagophore formation. In the following stages, the phagophore is elongated, and envelopes preselected cargo forming an autophagosome. In the course of the degradation stage, the autophagosome fuses with a lysosome, forming an autolysosome. During the degradation stage, a cell retrieves amino acids and other precursor molecules from degraded proteins and removes damaged structures that may disturb its homeostasis. Every stage of autophagy requires specific enzymes, proteins, and cofactors to occur. Recent studies indicate that lipid signaling is a vital part of a cellular pathway involved in autophagy, though an underrepresented one [9]. Identifying lipids involved in cellular signaling proves to be more difficult than other substances, such as proteins. Interestingly, recent technical advances allow us to appreciate their role more than ever.

## Significance of autophagy in pathology

Many studies uncovered dysfunctional mechanisms of autophagy occurring in various diseases. Neurodegenerative disorders such as Alzheimer’s disease (AD) and Parkinson’s disease (PD) are most often cited as having impaired autophagy [10]. This is because post-mitotic cells, such as neurons, are more prone to accumulate dysfunctional organelles and proteins. Further research found that the list of diseases involving autophagy is much extended [11]. Furthermore, the influence of autophagy on some diseases seems to be more complex than simply propagating the evolution of disease or preventing its development [12]. The most commonly mentioned disease in which autophagy has a dualistic role is cancer, where autophagy is often referred to as a double-edged sword [13]. Autophagy, a home-keeping mechanism, allows cells exposed to mutagens to recover. Dysfunctional autophagy allows damaged proteins and organelles to accumulate, propagating the genome instability and promoting genome mutation and possibly carcinogenesis [14]. On the other hand, autophagy is a known factor that allows cancer cells to grow and reproduce despite highly unfavorable conditions within the tumour [15]. Many studies indicate that cancer cells are more autophagy-dependent than noncancerous ones [16]. Other diseases involving autophagy include heart, muscle, liver (liver fibrosis and cirrhosis in particular), autoimmune, cardiovascular and inflammatory diseases [17–20]. Recent studies also indicate the role of autophagy in obesity and diabetes mellitus [19, 21]. Furthermore, autophagy also seems to have a profound role in aging and immune response to both bacterial and viral infections [22, 23]. Accordingly, further research investigating such a complex process might contribute to uncovering possible strategies to enable curative therapeutic opportunities.

### Regulatory mechanisms and molecular interactions during initiation

As mentioned above, the process of autophagy itself may be divided into 5 stages: initiation, elongation, maturation, fusion, and degradation. All of these stages combined were briefly discussed, but each of them deserves a separate description of its own [24].

The induction of autophagy is strictly linked to AMPK (AMP-activated protein kinase) and mTORC1 (mammalian target of rapamycin kinase complex 1) [25]. Both react to different conditions occurring in the cell and allow autophagy to proceed. When the cell is in nutrient-sufficient conditions, mTORC1 phosphorylates ULK1/2 (UNC-51 like kinase 1/2) and stops the induction of autophagy [26]. mTORC1 is activated by growth factors, amino acids and inhibited by starvation, ROS, and stress [27]. AMPK is an energy-sensing kinase that responds to the rise in AMP:ATP and ADP:ATP ratios and DNA damage [28]. AMPK has a dualistic role in the induction of autophagy. It inhibits mTORC1 and directly activates ULK1/2 complex. ULK1/2 complex is comprised of ULK1/2, Atg13, FIP200 and Atg101 proteins and is essential for the further progression of autophagy [29]. Atg (autophagy-related) proteins are a large family of proteins that oversee the correct course of autophagy at all stages. Over 30 Atg proteins have already been identified. ULK1/2 activates other proteins vital for autophagy induction, such as Beclin1, VPS34, and AMBRA1 [30]. VPS34 is a phosphatidylinositol 3-kinase (PI(3)K) that phosphorylates the 3 positions of phosphatidylinositol (PI), generating PI3-phosphate (PI3P) [31]. PI3P is a member of the phosphoinositide lipids family that has a significant role in many cellular processes. During autophagy, PI3P mediates many crucial Atg proteins’ binding to the surface of the phagophore, allowing them to work properly [32]. Beclin1 is known to have an important role in the nucleation of phagophores and maturation of the autophagosomes, which occur in further stages of autophagy. An important role during autophagosome formation is that of ER-mitochondria contact sites located in mitochondria-associated membranes (MAMs). ER-mitochondria contact sites provide a platform for the assembly of autophagosome initiation complexes [33–35]. Additionally, MAMs are also involved in the regulation of calcium signalling. Calcium flux between the ER and mitochondria influences various cellular processes, including autophagy. It was noted that appropriate calcium levels at the MAMs are essential for the initiation and progression of autophagy [33].

Beclin1 forms a protein complex with VPS34 and VPS15/p150 (Beclin1-VPS34-VPS15 complex), which is required for omegasome formation [36]. Omegasome is a PI3P-rich site in the ER (endoplasmic reticulum), where phagophores originate from [37]. A phagophore (sometimes referred to as an isolation membrane) is a double-membrane structure that expands during the elongation stage and eventually, as an autophagosome, envelops cargo intended for degradation in lysosomes [38].

**Fig. 1:**
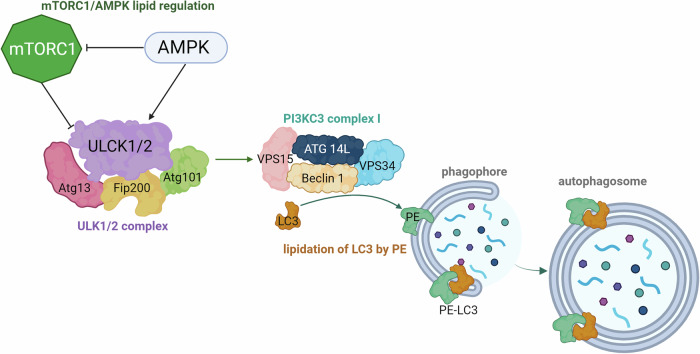
Schematic overview of autophagy induction with a focus on lipid involvement in signaling regulation. mTORC1, a key suppressor of autophagy, is inhibited by AMPK under conditions promoting autophagy. AMPK also activates the ULK1/2 complex, which consists of ULK1/2, Atg13, FIP200, and Atg101. Upon activation, the ULK1/2 complex stimulates the PI3KC3 complex I, composed of VPS15, Beclin1, VPS34, and ATG14L. This complex regulates the production of phosphatidylinositol-3-phosphate (PI3P) on the phagophore, a crucial lipid that signals the progression of autophagy. Further downstream, lipids play an essential role in conjugating phosphatidylethanolamine (PE) to LC3, facilitating the formation of the autophagosome. The conjugation of LC3 with PE ensures membrane expansion and curvature necessary for autophagosome maturation, emphasizing the critical roles of lipids in both signal propagation and structural development during autophagy.

Beclin1-VPS34-VPS15 is a core of PI3KC3 complexes (class III phosphoinositide-3-kinase complexes). PI3KC3 complex I (PI3KC3-C1) is formed with ATG14L and has a significant role in phagophore formation and cargo selection, while PI3KC3-C2 (complex II) is vital in further stages of autophagy [39]. Downstream events of PI3KC3-C1 activity result in the formation of LC3-PE, an autophagic cargo tether, formed by the conjugation of LC3-I (microtubule-associated protein 1A/1B-light chain 3) protein and the PE (phosphatidylethanolamine) lipid [40]. The conjugation is supervised by Atg7 and Atg3, which are ubiquitin-like enzymes [41]. Researches proved that PI3KC3-C1 activity is necessary for this process, as kinase-deficient mutants lack the required lipidation function [42]. The LC3-PE is then recruited to the autophagosome membrane. LC3-PE has a vital role in cargo selection since p62, with cargo attached, binds to LC3 directly [43]. p62 is also known as sequestosome 1 (SQSTM 1) and is a multifunctional protein with 2 autophagy-related domains. The first one is ubiquitin-associated domain (UBA), and the other is LC3-interacting region (LIR) [44]. Thereby LC3-PE and p62 interaction lies at the heart of selective autophagy. LC3-PE is degraded during the fusion of autophagosome and lysosome and is a viable way of monitoring cellular autophagic activity since it is detectable by immunoblotting, immunoprecipitation, and immunofluorescence [45]. The initiation of autophagy and LC3-PE formation is summarized in Fig. 1.

### Autophagosome development and cargo processing during elongation, maturation, and degradation

Phagophores undergo elongation, during which the double membrane expands into, eventually, an autophagosome. The major source of lipids required for phagophore expansion is the ER. The latter is connected to the phagophore through membrane contact sites (MCSs), allowing lipid transfer [46]. Responsible for this connection, as well as lipid transfer, are Atg9-Atg2-Atg18 complexes [47]. It has been established that Atg9 binds to the tips of phagophore and recruits Atg2 and Atg18 [47]. Atg18 possesses a PI3P-binding motif that is crucial for binding to phagophore extremities rich in PI3P and propagating the activity of Atg2 - a lipid transfer protein [48]. Once the complex is assembled, the phagophore expands and envelopes preselected cargo. Once the cargo is selected, the autophagosomes undergo maturation. During this process, Atg proteins dissociate from the surface of the autophagosome. One of the enzymes involved in this process is myotubularin 3 (MTMR3) - a PI3P phosphate [49]. MTMR3 dephosphorylates PI3P, which is required for efficient Atg proteins binding to phosphatidylinositol [50]. MTMR3 is recruited to autophagosomes by PI3P, and downstream events of its action promote maturation into autolysosomes. It has been established that the removal of PI3P from the surface of autophagosome destabilizes Atg proteins required in earlier stages and thus allows their recycling [9]. It is also important to note that the removal and Atg proteins make matured autophagosomes fusion-competent [51].

Another important regulator of maturation is the PI3KC3-C2 (complex II), which contains UVRAG instead of ATG14L [52]. Its role seems to be the activation of Rab7 GTPase, which propagates autophagosome maturation and is crucial for autolysosome formation during the degradation stage. Additionally, the PI3KC3-C2 is involved in many cellular pathways, including endocytic sorting.

**Fig. 2:**
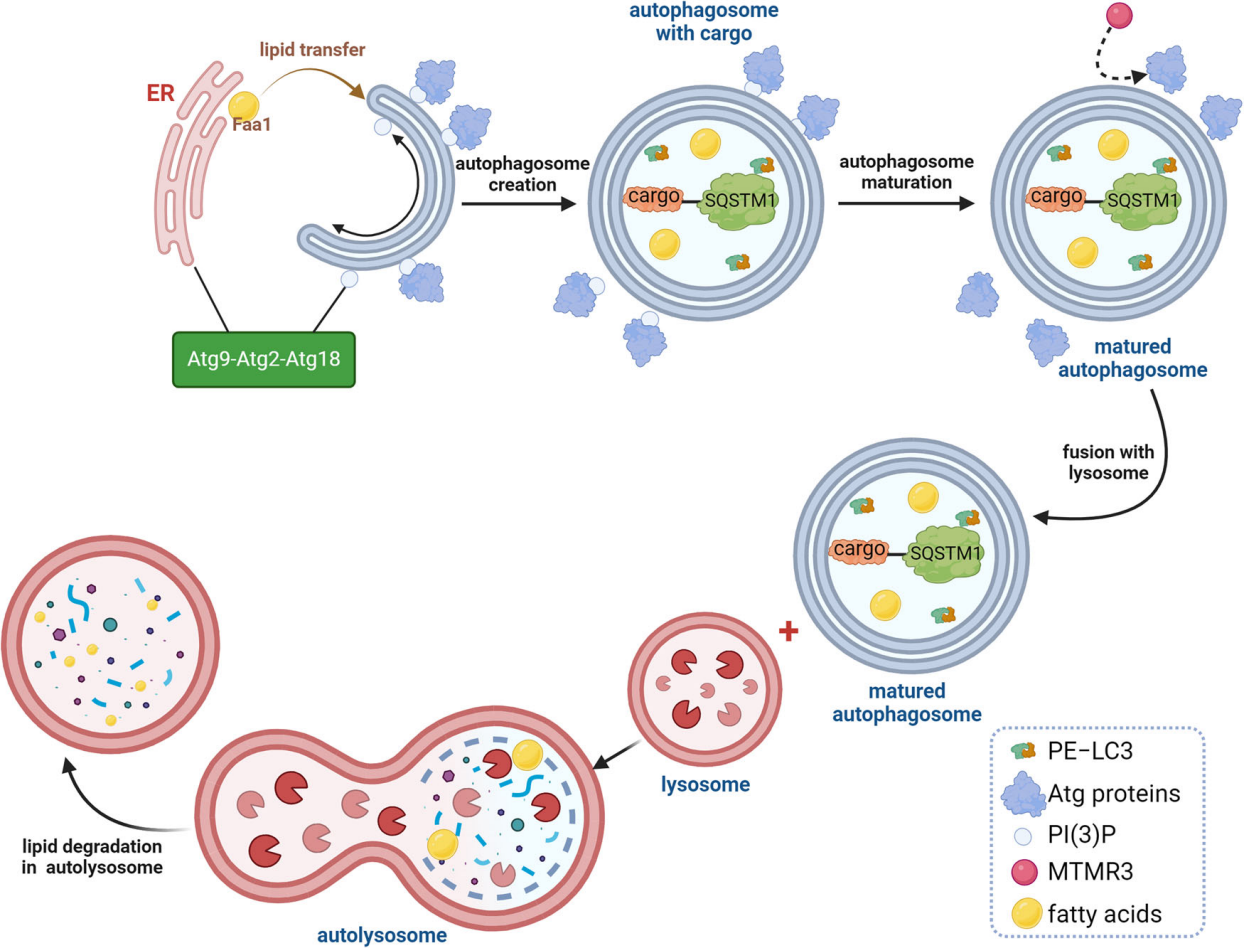
Schematic overview of the major steps of autophagy with a focus on lipid involvement. Atg proteins bind to PI3P on the phagophore surface, initiating the process. The Atg9-Atg2-Atg18 complex mediates lipid transfer from the ER to the growing phagophore, facilitating its elongation. Lipids play a critical role in regulating autophagy signaling at this stage. SQSTM1, with preselected cargo attached, binds to LC3-PE on the inner surface of the forming autophagosome. During autophagosome maturation, MTMR3 dephosphorylates PI3P, leading to the dissociation of Atg proteins, which is crucial for the autophagosome to become fusion-competent. Finally, lysosomes fuse with the matured autophagosome to form an autolysosome, where lipids and other cellular components undergo degradation in the final phase of autophagy.

During the degradation stage, the autophagosome fuses with a lysosome, and lysosomal hydrolases degrade its cargo. This event is controlled by Rubicon, UVRAG, SNAREs, Rab7, and other proteins. A schematic representation of the major steps of autophagy is summarized in Fig. 2.

## Lipid role in autophagy

Over the years, it became apparent that lipids play a major role in autophagy. As summarised above, lipids are involved in every step of autophagy, from induction to degradation. They regulate pathways converging on mTOR and thereby directly interfere in autophagy induction. Lipids, and PI3P in particular, also function as membrane-bound signaling entities responsible for the recruitment of proteins required for membrane dynamics. Furthermore, the binding of PE to LC3-I allows a stable connection of these crucial proteins with the membrane and, thus, is vital for membrane expansion and closure. A significant amount of studies suggest the involvement of lipid signalling and lipid metabolism disturbance in a large variety of diseases, e.g., neurodegenerative disorders, non-alcoholic fatty liver disease, and cancer [19].

### Phosphoinositides

Phosphoinositides are a group of phospholipids originating from the phosphorylation of the third, fourth, and fifth positions of the inositol group of phosphatidylinositol. The phosphorylation results in seven derivatives that a group of phosphoinositide kinases and phosphatases can interconvert. Even though they represent a small percentage of phospholipids, their role is far from insignificant. They are involved in membrane dynamics, cellular signaling and many other processes important for this review, involving autophagy [53]. Their role is best defined in the initiation of autophagy, autophagosome formation and maturation. Phosphoinositides are strictly connected to the mTORC1’s role in the induction of autophagy. As a response to insulin, growth factors, and general availability of nutrients, the class I phosphoinositide 3-kinase phosphorylate PI2P (phosphatidylinositol 4,5-bisphosphate) to PI3P (phosphatidylinositol 3,4,5-triphosphate) [9]. PI3P then activates PDK1 (phosphoinositide-dependent kinase-1), which phosphorylates Akt (also known as protein kinase B) [54]. The downstream events of Akt activation lead to the activation of mTORC1 and, consequently, the inhibition of autophagy [55]. On the other hand, when a cell is exposed to general nutrient-deficient conditions, PI3P is dephosphorylated by PTEN (phosphatase and tensin homolog, a 3-phosphatase) [56]. This causes the downstream events that result in mTORC1 inhibition and, thus, autophagy induction. Interestingly, PTEN is considered a tumor suppressor with growth regulatory functions [56]. Lipids involvement in autophagy is summarized in Table 1.

**Table 1:** Summary of lipids involvement during autophagy.

Phosphoinositides	Sphingolipids
Autophagy induction	Autophagy induction
Autophagy inhibition	Autophagy inhibition
Atgs recruitment	Beclin1-VPS34-VPS15 activation
Phagophore creation	Phagophore elongation
Autophagosome maturation	PE precursors
Present in autophagosomal membranes	Present in autophagosomal membranes

During maturation, the PI3P dephosphorylation by various phosphatases makes autophagosomes fusion-competent [57]. For example, it has been established that the depletion of MTMRs phosphatases results in autophagosome accumulation, which is associated with many diseases [58]. Another phosphatase that targets PI3P during the maturation stage is Synj1 (synaptojanin 1) known for its function in synaptic vesicle endocytosis and autophagy [59]. Mutations (R258Q and R459P) within SAC1 domain result in the accumulation of immature autophagosomes in human neurons [60, 61]. These mutations are known to contribute to the pool of the early onset of PD.

### Sphingolipids

Sphingolipids are a class of lipids that are involved in many cellular processes, including apoptosis, proliferation and autophagy. They are also found in the membrane of eukaryotes and participate in membrane dynamics. Among many sphingolipids, ceramide (Cer), sphingosine-1-phosphate (S1P) and dihydroceramide (dhCer) are most often cited as having a role in autophagy [62]. Sphingolipids, and ceramide, in particular, are found in autophagosomal membranes [63]. The available studies revealed that sphingolipids formed de novo in ER might be the main lipids transported during the lipid transfer of the elongation stage and, thus, autophagosome formation is possible [64, 65].

Ceramide is involved in the induction of autophagy in both mTORC1- and AMPK-dependent manner. Cer inhibits Akt activation, suppressing the action of mTORC1 [66]. It also causes the downregulation of nutrient transport proteins, leading to the AMPK stimulation [67]. Furthermore, there was noted, that Cer propagates the dissociation of Beclin1 from the Beclin1/Bcl-2 complex, which is a prerequisite for Beclin1-VPS34-VPS15 formation [62]. The exact interaction is yet to be discovered, but it can be assumed that Cer has a significant role in autophagy induction via at least 3 different pathways. Studies conducted on cultured cell lines revealed that Tamoxifen’s increase of endogenous Cer levels (an estrogen receptor antagonist used widely in breast cancer) promoted autophagy-associated cell death, which theoretically synergizes with its anti-tumor action [68].

S1P is formed in two steps from ceramide by ceramidase converting it to sphingosine, which is then phosphorylated to S1P by sphingosine kinase 1 and 2 (SK1 and SK2). In studies conducted on cells expressing an inactive form of SK1, the autophagy is blocked, which indicates that both S1K and S1P are required for autophagy induction. Furthermore, cells overproducing S1K have increased levels of autophagy. This interaction can likely be explained by the influence of S1P on phagosome formation. Increased levels of S1P stimulate sphingosine-1-phosphate lyase 1 (SGPL1), which breaks down S1P into phosphoethanolamine (EAP) and hexadecanal. EAP can be converted into CDP-ethanolamine by a cytidylyltransferase (PCYT2), and then to PE by a methyltransferase. As stated above PE and LC-3 interaction is an integral part of autophagy, and studies suggest that increased PE levels may propagate its course [69, 70]. Indeed, research proves that an extra supply of PE upregulates autophagy [71]. Interestingly, the administration of PE precursor ethanolamine extends the lifespan of yeast, flies and some mammalian cells [71]. It has been suggested that S1K inhibits mTORC1 by pathways different than Akt, but the exact interaction remains elusive. It is important to note that S1P is considered a tumor-promoting lipid due to its impact on autophagy, and increased S1P levels have been identified as a drug-resistance mechanism in some cancer types. dhCer was recently identified as another sphignolipid-derivative involved in autophagy [72]. It was demonstrated that its elevated levels stimulate both autophagy and autophagy-associated cell death. Endogenously added dhCer promoted autophagy in gastric and colon cancers [73], and its intracellular accumulation by anticancer drugs (such as fenretinide and gamma-tocotrienol) was attributed to causing cancer cell death [74].

Sphingomyelin is another sphingolipid connected to autophagy. Sphingomyelin accumulation occurs in Niemann-Pick diseases (NPD) type A and B and causes severe disturbance in cell homeostasis [75]. Corcelle-Termeau et al. indicated that sphingomyelin in NPD patients acts as a negative regulator of autophagy, hindering the organization of autophagosomes [76].

## Autophagy and lipotoxicity

The role of autophagy in lipotoxicity involves the cellular process playing a critical role in mitigating the harmful effects of lipid accumulation and lipid-induced stress within cells. Moreover, autophagy plays a role in regulating lipid metabolism by influencing processes such as lipogenesis, lipolysis, and fatty acid oxidation. This dynamic interplay helps cells adapt to changes in nutrient availability and maintain lipid homeostasis [77]. The adipose tissue responds to excess nutrients by increasing their uptake and storage in existing adipocytes or by expanding their number through adipogenesis [78]. However, according to the adipose tissue expandability hypothesis, every individual has a limited margin at which the adipose tissue can expand [79]. This is determined *inter alia* by environmental and genetic factors. Once the limit is reached, lipids begin to accumulate in “ectopic” non-adipose tissues [80]. The lipids accumulate in β-cells, hepatocytes, neurons, skeletal myofibres and cardiomyocytes with limited lipid storage abilities. Lipotoxicity is defined as a deleterious metabolic syndrome occurring when non-adipose cells are unable to store excess lipids in LDs. The lipotoxic effects of lipid accumulation include disturbances in metabolism, oxidative stress, mitochondrial dysfunction, proinflammatory signaling and accumulation of lipid derivatives like diacyloglycerol and ceramide [81]. LDs are organelles that store lipids in the form of triacylglycerols, cholesterol, and retinal esters confined in a monolayer of phospholipids. Their membrane is coated with proteins called perilipins that play a vital role in the metabolism of LDs. LDs’ size varies depending on the type of cell that contains them. More importantly, lipids inside LDs can be utilized by the cell for different purposes, such as energy provision and cell membrane component synthesis. LDs function as storages of free fatty acids and sterols that can be accessed by a cell through lipolysis and lipophagy [82]. The size of the LDs is a factor that conditions whether an LD (a) is targeted by microautophagy and digested piece by piece as parts of larger vesicles are pinched off and trapped inside of double-membrane vesicles or (b) is targeted by macroautophagy and digested in lysosomes as a whole small vesicle [82]. Both of these types of autophagy lead to a breakdown of those lipids by lysosomal acid lipases that catabolize triacylglycerides, diacylglycerides, cholesteryl and retinyl esters [83]. Interestingly, the recent studies focusing on LDs, involve compounds interacting with both LDs and the key autophagosome protein LC3, which may enhance the autophagic degradation of LDs. This novel approach targets LDs through autophagy, which seems reasonable and could potentially be extended to other cellular structures or biomolecules, both protein and non-protein, broadening the scope of applications for this strategy in the development of novel therapeutic interventions [84]. Considering lipotoxicity and its ramifications on cellular processes, particularly autophagy, several critical knowledge gaps can be identified that, if addressed, could significantly advance the understanding of these complex mechanisms. Firstly, the precise molecular pathways by which different lipids contribute to lipotoxicity-induced autophagy remain inadequately defined. Shina et al. described that autophagy might moderate and protect against hepatic lipotoxicity, particularly induced by oxidative stress [85]. In this scenario, autophagy, including its specialized form of mitophagy, acts as a cellular defense mechanism. Mitophagy, specifically, is the process by which cells selectively degrade and recycle damaged mitochondria. When lipotoxic conditions impair mitochondrial function and increase ROS production, mitophagy is upregulated as a protective response. By removing the damaged mitochondria, mitophagy prevents further oxidative stress and the potential onset of lipoapoptosis, a form of cell death triggered by excessive lipid accumulation [86–88],. The process of mitophagy can play a critical adaptive response that cells employ to mitigate the damaging effects of lipid overload and maintain cellular homeostasis. This highlights the essential role of autophagy and mitophagy in protecting cells from the detrimental impacts of lipotoxic conditions, underscoring their importance in cellular health and disease prevention. Thus, the understanding the specific signaling cascades and lipid-protein interactions at play could unveil targeted intervention points for therapeutic development. It seems crucial the role of lipid droplets (LDs) in sequestering toxic lipids and their subsequent impact on autophagy induction. In the other study suggested, that LDs biogenesis serves as a universal cellular response to times of increased autophagic activity, offering a lipid buffering system to counteract lipotoxic damage within cells [89]. This could elucidate how cells balance energy storage and detoxification, providing insights into metabolic diseases.

Furthermore, the differential impact of various fatty acid species on the autophagy machinery remains a significant area for exploration. Identifying how unsaturated versus saturated fatty acids influence autophagic processes could refine dietary guidelines and pharmacological strategies to mitigate lipotoxicity.

### Oxiapoptophagy and oxysterols

Oxiapoptophagy was defined as a cell death induced by some oxysterols, including 25-hydroxycholesterol (25-HC), 7β-OHC, 19-hydroxycholesterol, cholesterol 5α, 6α epoxide and 25-hydroxycholesterol [90]. It was observed that cytotoxic concentrations of these oxysterols can provoke cell death with symptoms related to oxidative stress, apoptosis and autophagy, thus, new cell death pathways were suggested [90, 91]. It is known that oxysterols are oxidized derivatives of cholesterol. Besides, c.a. 60 various oxysterols can be produced during the oxidation process of cholesterol and can simulate pro-apoptotic and pro-autophagic signals [92]. Seo et al. showed that 25-HC induced oxiapoptophagy in osteoblasts, which highlighted its crucial role in osteoporosis [93]. A similar effect of 7α,25-DHC was demonstrated on chondrocytes, where caspase-related cell death through extrinsic and intrinsic apoptotic mechanisms was observed [94]. 7KC, 7β-OHC and 24S-OHC trigger oxiapoptophagy symptoms including ROS release, casp-3 activation, PARP degradation and Bcl-2 suppression. There was noted that increased concentrations of these oxysterols are observed in brain lesions. However, Nury et al. observed that this process can be inhibited by α-tocopherol [91]. It is also crucial to mention the role of omega-3 and omega-9 fatty acids in preventing autophagy in oxysterol-induced oxiapoptophagy. Oleic acid (C18:1 n-9) and docosahexaenoic acid (DHA, C22:6 n-3), which are major components of the Mediterranean diet, exhibit anti-lipotoxic and anti-autophagic properties. This significant observation underlines the importance of considering the impact of lipid mixtures on autophagy, as the ratio between different types of lipids can influence the induction or suppression of autophagy. Notably, fatty acids themselves, aside from their receptors (PPAR), have not been cited in the review, underscoring a critical area of exploration. References highlighting these findings include studies by Debbabi et al. [95] and Nury et al. [91], which demonstrate the protective effects of oleic acid and DHA against 7-ketocholesterol-induced oxiapoptophagy in cellular models. Consequently, this means that the cellular lipidome’s composition - reflecting the types and amounts of lipids present - can effectively switch autophagy on or off [96]. By modulating the lipid composition or altering specific lipid ratios within cells, it might be possible to control autophagy more precisely, offering a novel strategy for treating conditions like neurodegenerative diseases, cancer, and metabolic disorders.

### Peroxisome proliferator-activated receptors family signaling

Peroxisome proliferator-activated receptors (PPARs) are nuclear receptors that act as transcription factors. They regulate gene expression and influence various processes such as cell differentiation, metabolism, and tumor development [97]. There were identified three main groups (isoforms) of PPARs: alpha(α), beta/delta(β/δ), and gamma(ɣ)- each expressed in certain types of tissues [98] (Table 2). To function as a transcription factor, PPARs must bind to Retinoid Receptor X(RXR) and a lipid ligand that is nowadays more identified explicitly as free fatty acids such as oleic acid and linoleic acid, prostaglandins, and other eicosanoids. PPARs use their ligand-binding domains to bind to these ligands. PPARs are proteins that are transcribed by different genes on three different chromosomes. PPARα genes loci are 22q12-13.1, PPARβ/δ is located in 6p21.2-21.1 and PPARɣ is located in 3p25(there are 4 isoforms created through alternative splicing [99]. Genetic changes in these locations result in loss of function and have their representation in individuals’ phenotype. Lipodystrophy, insulin resistance and acanthosis nigricans have been described as associated with mutations of these genes [100]. There are also polymorphisms concomitant with obesity and mutations that decrease insulin resistance risk [101]. Organ expression of PPAR types is summarized in Table 2.

**Table 2:** Organ expression of different PPAR types [50].

PPARα	PPARβ/δ	PPARɣ
Liver Brown adipose tissue Heart Kidneys	Colon Small intestine Liver Heart Lung Brain	Adipose tissue Intestine Macrophages

PPARα expression is focused mainly on hepatocytes, brown adipose tissue, heart and kidneys. It has been proven that it is involved in catabolism and the uptake of fatty acids by promoting the expression of genes that influence the transport and binding of fatty acids. A correlation with autophagy was discovered during studies on tuberculosis-infected macrophages where PPARsα were responsible for the maturation of autophagosomes and, thus, stimulation of autophagy [102]. The same study underlined that activated PPARα leads to activation of Transcription Factor EB (TFEB) that stimulates the expression of a series of genes that are key to autophagy and lysosomal degradation. Together PPARα and TFEB act as transcription factors that enable autophagy through stimulation of the expression of genes. PPARβ/δ are expressed mainly in the colon, small intestine, liver, heart, lung, and brain. PPARsβ/δ were described as impactful in a group of society-haunting diseases such as obesity, atherosclerosis, diabetes, and cancer [98, 99]. On the other hand, PPARɣs, principally active in adipose tissue, the intestines, and macrophages, the function is described in glucose uptake, formation of adipose tissue and fatty acids storing [98, 99]. PPARɣ is clearly involved in the process of nutrient distribution control and thus have a direct connection to autophagy and cancer treatment strategies [103]. Through inducing PTEN and in a result, inhibition of PI3K PPARɣ decreases the amount of PIP3 in a cell. A lower concentration of PIP3 determines inhibition of mTOR pathway, thus inducing autophagy. PPARɣ has been proven to induce autophagy in colorectal and breast cancer [104, 105].

PPARs and several other systems regulate lipophagy at the highest level by controlling downstream factors to ensure that free fatty acids levels in a cell are optimal [106] according to a general rule that states that nutrient starvation stimulates autophagy and their abundance inhibits the process.

### Mitophagy

Mitophagy is a process of removing damaged mitochondria from the cell through macroautophagy. Mitochondria are organelles that regulate cellular energy metabolism and cellular death. Mitochondria are also producers of reactive oxygen species (ROS) and an origin of damage-associated molecular patterns (DAMPs) such as mitochondrial DNA (mtDNA) that, when located in cytosol stimulate inflammatory responses. ROS’s presence is responsible for the oxidation of proteins, lipids, and nucleic acids, leading to cell malfunction. Damaged mitochondria are removed from the cell through autophagy in two steps. The first is the induction of general autophagy; the second stage is the process of marking damaged mitochondria for autophagic recognition. As reliable methods for studying the effect of mitophagy in mammalian cells are not yet available, mitophagy is best documented in yeast cells, although it was first observed in mammalian hepatocytes of a rat. Mitophagy is said to be a crucial part of the terminal differentiation of erythrocytes, paternal mitochondrial degradation, neurodegenerative diseases, and ischemia or drug-induced tissue injury [107]. Mitophagy is also considered important for age-related diseases and aging that are strongly connected to malfunctioning mitochondria. Studies have shown a connection between well-known beneficial health factors- physical exercises and mitophagy [108]. Mitophagy is proven to increase its pace when harsh conditions for the cell appear- hypoxia, oxidative stress, and starvation are those stimulating factors. On the other hand, impaired mitophagy is linked to many positive and negative processes, such as development, aging, cardiovascular diseases and cancer.

### Pexophagy

Pexophagy represents a discerning autophagic process characterized by selectivity in targeting and eliminating peroxisomes. It has proved to be vital for the homeostasis of those organelles, but still, little is known about the process itself. Peroxisomes are catabolic organelles that degrade large particles such as purines and fatty acids but also participate in anabolic processes such as the synthesis of bile acids. It is proven that peroxisomes generate ROS and RNS but also scavenge for them and play an important role in redox balance. Peroxisome biogenesis disorders (PBDs) have mainly genetic backgrounds and are caused by incorrect genesis, mainly their assembly, or functional defects of peroxisomes [109]. It is known that those disorders can affect different organs involving the brain, eyes, kidneys, liver, or adrenal cortex [109, 110].

## Autophagy-related lipids and their role in disease

Neurodegenerative diseases are one of the biggest challenges facing modern medicine. While their etiology and pathophysiology differ, they are most often attributed to the accumulation of dysfunctional proteins. Indeed AD, PD, Huntington’s disease (HD), amyotrophic lateral sclerosis (ALS) and frontotemporal dementia (FTD) all share the property of intracellular accumulation of proteins. Unfortunately, their common feature is also the lack of effective treatment. Research indicates that impaired autophagy may be a common link between these diseases, although exact mechanisms remain elusive. Since lipids are heavily involved in autophagy regulation and course, their role in neurodegenerative diseases is unquestionable.

### Parkinson’s disease

Early onset of PD is characterized by tremors, muscular rigidity, and hypokinesia occurring at a young age. Several genes predisposing to this condition have been identified, and the mutations R258Q and R459P of Synj1’s SAC1 domain are a recent addition to this pool. Patients with autosomal recessive mutations of Synj1 developed Parkinson’s symptoms during the third decade of life [111]. Research on knock-in flies with Synj1 mutations revealed the accumulation of immature autophagosomes, dopaminergic neuron loss, and general neurodegeneration, directly linking the impaired autophagy to PD [59].

### Amyotrophic lateral sclerosis

ALS is the most common form of motor neuron disease, in which motor neurons selectively degenerate [112]. Affected neurons accumulate misfolded and toxic proteins, leading to neurodegeneration. During the course of the disease, motor neurons in the brain and the spinal cord degrade, causing progressive muscle loss and eventually respiratory failure. Although the exact mechanism of ALS is unknown, recent studies underlined the significance of autophagy in misfolded proteins' clearance and suggested that impaired autophagy may be the basis for ALS onset [113]. During the studies with certain Atg genes knocked out, it has been established that Atg-deficient mice presented ALS-like phenotypes by 3-4 weeks of age [114]. Histological findings revealed a high degree of protein accumulation that correlated with neurodegeneration.

### Alcoholic fatty liver disease (AFLD)

Alcoholic fatty liver disease is caused by excessive alcohol consumption and is described as the destruction of the liver tissue by ethanol. The mechanism of inflicting damage to hepatocytes includes mitochondrial damage, oxidative stress and LDs accumulation that results in cell death. Liver steatosis (accumulation of lipids in the form of LDs in the liver tissue) is a particularly characteristic of alcohol abuse. It has been proven that short-term ethanol consumption stimulates lipophagy and mitophagy in hepatocytes, presumably acting as a repair mechanism to prevent steatosis and damage inflicted on mitochondria [106, 115]. Nonetheless, ethanol abuse and chronic use impair autophagy and thus lipophagy [116], probably by activation of mTOR signalling [117]. To conclude, lipophagy could be targeted as a process suitable to use for AFLD therapy as it is inhibited in alcohol-abusing patients [118].

### Non-alcoholic fatty liver disease

The term non-alcoholic fatty liver disease (NAFLD) is used to describe many liver conditions characterized by lipogenesis and accumulation of lipids in hepatocytes. Insulin resistance and usage of glucose molecules to synthesize not glycogen but lipids in the process of lipogenesis are involved in NAFLD. Most research suggests that lipophagy counteracts NAFLD progression and is inhibited in some instances of the disease [19, 106].

### Liver fibrosis

The term liver fibrosis describes end-stage liver diseases that lead to the formation of a disrupted liver structure with the emergence of scar tissue built of connective tissue and regenerative nodules. Those diseases are mainly AFLD and NAFLD but also viral infections like hepatitis B or hepatitis C, autoimmune diseases and other less common causes. The disrupted structure of the liver due to abundant extracellular matrix accumulation causes impairment of liver function and insufficiency of the organ itself. It has been proven that hepatic stellate cells play a vital role in liver fibrosis progression by secreting fibrogenic factors which stimulate collagen fibers synthesis [119]. Research showed evidence that lipophagy plays a part in the process of liver fibrosis. It has been shown that in human and mouse mice, autophagy induces stress on the endoplasmic reticulum of the stellate cell that induces fibrogenic activity [120]. Apart from endoplasmic reticulum stress, activation of stellate cells causes LDs contents to be released to the extracellular environment, oxidative stress, and accumulation of p62 and G proteins overexpression [106, 121]. Zhang et al. proved that antioxidants such as glutathione and N-acetyl cysteine inhibit autophagy, indicating the potential path for pharmacotherapy [122].

### Autophagy and lipophagy in heart and skeletal muscle

In the context of the heart and skeletal muscle, autophagy and lipophagy play critical roles in ensuring these tissues’ proper function and energy balance. Firstly, autophagy assists in removing damaged or misfolded proteins in both cardiac and skeletal muscle cells. This process contributes to the regulation of cellular energy balance by recycling cellular components during periods of nutrient deficiency or stress. Moreover autophagy, particularly mitophagy, maintains mitochondrial quality control, i.e. selectively removes damaged or dysfunctional mitochondria [123, 124]. It was observed that myocardial autophagy (diagnosed as autophagic vacuoles in cardiomyocytes) has the potential to enhance the prognosis of heart failure by impeding the advancement of myocardial degeneration [125]. In turn, lipophagy plays a role in the mobilization and utilization of stored lipids for energy production in muscles. In times of energy demand, the breakdown of lipid droplets through lipophagy provides fatty acids for mitochondrial oxidation. Lipophagy also helps to prevent lipotoxic effects by efficiently clearing residual lipid droplets. As a non-direct effect, dysregulation of lipophagy has been associated with various metabolic disorders, including also those affecting the cardiovascular system. Lam et al. investigated the potential role of autophagy in the reversal of lipid droplet deposition observed in skeletal muscle post-bariatric surgery in morbidly obese patients in in vitro model. Authors performed a simultaneous treatment with an autophagic inhibitor (bafilomycin) and activator (rapamycin), which unexpectedly led to decreased lipid accumulation, suggesting a novel pathway involving p62-mediated turnover of lipid droplets and the potential export of triglycerides into the extracellular space [126]. Similarly, Cui et al. blocked autophagy by mucolipin 1 (MCOLN1) and observed reduced oxidation and re-esterification of lipophagy-derived fatty acids. This emphasizes the significance of the established lysosomal fusion to the plasma membrane as the primary pathway for organizing these fatty acids. Additionally, the efflux, reuptake, or extracellular trafficking of fatty acids might be crucial in facilitating cell-to-cell lipid exchange and signalling [127].

### Autophagy in age-related macular degeneration

Age-related macular degeneration (AMD) is a frequent neurodegenerative disease among age-related diseases, leading to blindness in the elderly [128, 129]. Currently, there is no effective treatment available for it. However, a better understanding of autophagy and the role of lipids in the retina can lead to new therapeutic approaches. It was indicated that lipid metabolism involving apolipoprotein E (APOE) plays a crucial role in lipid trafficking in AMD. Authors observed that AMD’s retinal pigment epithelium (RPE) contains less dense cytoplasm, more lipid droplets, increased glycogen granules, and enlarged autophagosomes [129]. There have been found connections between AMD and mutations in various genes related to cholesterol, such as APOE, LIPC, CETP, and ABCA1. People who inherit the ɛ2 APOE allele are at a higher risk of developing the disease. APOE plays an important role in transporting lipids across cell membranes and is highly expressed by the RPE [130, 131].

Another study has suggested that oxysterols are involved in many age-related diseases, including both non-tumoral and tumoral ones. The study also found that oxysterol-induced cell death, oxidation, and inflammation could contribute to aging [132]. Thus, oxidized fatty acids, proteins, and lipoproteins may play significant roles in the pathogenesis of AMD. It has been observed that high oxygen levels and active fatty acid metabolism can cause the degeneration of RPE cells and the progression of AMD [133]. According to the available studies, targeting lipid metabolism in AMD seems a promising strategy to restore lipid processing in the RPE and future AMD therapies.

### Recent advances in lipid-autophagy research

Recent discoveries involve lipid sensors such as oxysterol-binding proteins (OSBPs) and underline their role in modulating autophagy. Oxysterol-related proteins (ORPs), including OSBP-related proteins, facilitate the exchange of lipids like cholesterol and phosphatidylinositol 4-phosphate (PI4P). This exchange is crucial for the formation and maintenance of autophagosomes, which are responsible for sequestering and degrading selective cargo during autophagy [134, 135]. The available studies indicate OSBPs’ influence on lipid metabolism and autophagy in a coordinated manner, affecting cell survival and disease progression. Tu et al. [136] have identified OSBPL7 and OSBPL11 as new lipid transfer proteins essential for the selective cargo in macroautophagy. In the other study was presented a significant role of lipid droplets in the formation of autophagosomes and their selective degradation through lipophagy, contributing to cellular energy balance. Interestingly, in this process syntaxin18 (STX18) revealed functions as a negative regulator, binding ATG14 and disrupting its interaction with ATG8 family proteins, which inhibits the formation of the PI3KC3-C1 complex and, consequently, lipophagy. When STX18 is knocked down, ATG14-mediated lipophagy is activated, leading to the degradation of lipid-associated proteins like Viperin, which has implications for lipid metabolism and viral replication [137].

Recent advances also demonstrate the development of small molecules that target lipid-autophagy pathways. Zhang et al. indicated several autophagy-associated targets, including AMPK, mTORC1, ULK1, IMPase, LRRK2, beclin-1, TFEB, GCase, ERRα, C-Abelso, that have shown promise in modulating autophagy in Parkinson’s disease [138]. In the same disease has been identified estrogen related receptor α (ERRα), as a key regulator in aggrephagy by inhibiting autophagosome formation. Aggrephagy is another specialized form of autophagy that targets misfolded or damaged protein aggregates for degradation. It was noted that ERRα inverse agonist XCT 790 has been shown to clear α-synuclein aggregates in an autophagy-dependent, mTOR-independent manner, offering potential neuroprotective effects in Parkinson’s disease models by enhancing autophagosome biogenesis [139]. Using a high-content phenotypic assay, researchers identified small molecules such as RH1115, which modulates autophagic flux by targeting proteins like Lamin A/C and LAMP1, promoting neuronal health and altering lysosome positioning for potential therapeutic benefit [140]. In the other study proposed molecules aiming for selective autophagy receptors (SARs) and related proteins, which can stop or reverse the disease progression in cancers, inflammation, neurodegeneration, and metabolic disorders [141]. Thus, the current developments highlight the intricate role of lipid-autophagy pathways in disease regulation, revealing lipid sensors and lipid exchange mechanisms that are critical for autophagosome formation and function, as well as small molecules that modulate these processes, offering potential therapeutic strategies for neurodegenerative diseases, cancer, and metabolic disorders.

### Clinical implications in autophagy-related diseases

The available data suggest that targeting autophagy pathways can be promising due to restoring lipophagy activity, which has been proposed e.g., as a therapeutic strategy for NAFLD treatment [142]. Additionally, several studies indicated that small molecules that enhance lipophagy, such as resveratrol and spermidine, are being explored in clinical trials for their ability to alleviate hepatic lipid accumulation [143–145]. Recent pre-clinical studies used ceramide-based therapies based on CerS inhibitors in combination with standard-of-care treatments to induce autophagic cell death in glioblastoma. This approach represents a promising avenue for targeting lipid metabolism pathways in autophagy to enhance cancer therapy [146]. Interestingly, clinical studies show that patients with GBA1 mutations have an increased risk of developing Parkinson’s disease. Therapies aimed at restoring glucocerebrosidase activity, such as ambroxol, are currently in trials to enhance autophagy-lysosomal function and reduce neurodegenerative progression [147]. There was also suggested that enhancing macrophage autophagy to clear lipid droplets has been proposed as a therapeutic strategy to prevent plaque progression [148]. Experimental treatments utilizing rapamycin, which activates autophagy, are under investigation to determine their potential to reduce atherosclerotic plaque burden in patients with cardiovascular disease [149]. The study performed on animal models investigating autophagy-enhancing drugs, such as trehalose, showed that it can restore lipid homeostasis and autophagic flux in neurodegenerative diseases. Thus, such therapies may help to reduce the accumulation of toxic proteins and mitigate cognitive decline [150]. Interestingly, in one study, the authors used metformin, which activates AMPK and autophagy, and it was studied in diabetic patients to improve insulin sensitivity and lipid metabolism. Metformin demonstrated here that restoring autophagic activity in adipocytes was explored as a treatment for metabolic syndrome [151].

Thus, as was described above, targeting autophagic pathways might be effectively implemented in therapeutic procedures for various diseases.

## Conclusions

Autophagy represents a complex cellular process that balances between promoting cell survival and facilitating cell death. Its integral role in the pathogenesis of various diseases has been extensively explored, driving forward our understanding of mechanisms underlying conditions that remain challenging to treat. The intricate investigation into the role of lipids in this process explains the profound complexity of autophagy and heralds the potential for groundbreaking advancements in the treatment of diseases and preventive healthcare. Lipids, far from merely serving as structural components, play a proactive role in coordinating autophagic pathways, thereby revealing novel avenues for targeted pharmacological interventions.

The findings highlight the importance of considering the dynamic interplay between different types of lipids and their ratios in modulating autophagy. Particularly, the discovery that specific lipid compositions can either induce or inhibit autophagy adds a critical layer to our comprehension of this cellular mechanism. Such knowledge clarifies our understanding of cell biology and paves the way for developing innovative therapeutic strategies that leverage these lipid-autophagy interactions. Given the significant impact of dietary lipids on the modulation of autophagy, there arises a compelling argument for exploring nutritional approaches alongside pharmaceutical ones in disease management and prevention. Recognizing the significant role lipids play in autophagy underlines the undeniable need for more dedicated research. The field of research focusing on the interaction between lipids and autophagy is broad and filled with opportunities to develop.
